# MoCA-screen-positive status differs across intraocular pressure subtypes of primary open-angle glaucoma

**DOI:** 10.3389/fmed.2026.1912812

**Published:** 2026-09-24

**Authors:** Rao Fu, Yiyan Wang, Guoping Qing

**Affiliations:** Department of Ophthalmology, Beijing Tongren Hospital, Capital Medical University, Beijing, China

**Keywords:** cognitive impairment, high-tension glaucoma, hypotension, Montreal cognitive assessment, normal-tension glaucoma, primary open-angle glaucoma, retinal nerve fiber layer

## Abstract

**Background:**

Cognitive vulnerability may differ across intraocular pressure-defined subtypes of primary open-angle glaucoma (POAG), but visually dependent cognitive-screening tasks and residual confounding complicate interpretation.

**Methods:**

This single-center observational study included 93 patients with POAG [49 normal-tension glaucoma (NTG) and 44 high-tension glaucoma (HTG)]. A corrected Montreal Cognitive Assessment (MoCA) score <26 defined MoCA-screen-positive status. A vision-reduced MoCA-22 score was calculated by removing the eight points assigned to visuospatial/executive and naming tasks. Sequential NTG-vs.-HTG models added demographics, visual field index (VFI), retinal nerve fiber layer (RNFL) thickness, diabetes, and systolic blood pressure. Subtype-by-PSQI and subtype-by-RNFL interactions, strict peak-IOP classification, structure-function correlations, mood-adjusted PSQI models, and bootstrap internal validation were examined.

**Results:**

MoCA-screen-positive status occurred in 44/93 participants (47.3%) and was more frequent in NTG than HTG (59.2% vs. 34.1%; crude OR: 2.80, 95% CI: 1.20–6.52; *P* = 0.017). The association remained in the fully adjusted sequential model (OR: 8.47, 95% CI: 1.81–39.61; *P* = 0.007) and after excluding six HTG participants with peak IOP <25 mmHg (OR: 6.74, 95% CI: 1.44–31.41; *P* = 0.015). Vision-reduced MoCA-22 was lower in NTG [18 (16, 19) vs. 19 (17.75, 20); *P* = 0.043], including after full adjustment (beta = −0.88, 95% CI: −1.63 to −0.14; *P* = 0.020). Subtype interactions were not significant for PSQI (*P* = 0.604) or RNFL (*P* = 0.082). In exploratory subtype models, study-defined history of low blood pressure study-defined history of low blood pressure (<110/<65 mmHg; recorded only among NTG participants), higher PSQI, and thinner RNFL were associated with screen-positive status in NTG; thinner RNFL was associated in HTG. Apparent AUCs were 0.940 (95% CI: 0.869–0.988) for NTG and 0.892 (0.763–0.989) for HTG; bootstrap-corrected AUCs were 0.900 and 0.851.

**Conclusion:**

NTG was associated with more frequent MoCA-screen-positive status and lower vision-reduced MoCA scores after measured visual, vascular, and metabolic adjustment. The subgroup correlates and model performance remain exploratory and require prospective external validation; the present data do not establish distinct causal NTG and HTG phenotypes.

## 1 Introduction

Primary open-angle glaucoma (POAG) is a chronic optic neuropathy characterized by progressive retinal ganglion cell loss, retinal nerve fiber layer (RNFL) thinning, and corresponding visual-field damage (1, 2). Although intraocular pressure (IOP) remains the principal modifiable risk factor, glaucoma is increasingly viewed as a complex neurodegenerative disorder in which ocular, vascular, and systemic susceptibility factors interact (3–5). This broader view is particularly relevant to normal-tension glaucoma (NTG), in which typical glaucomatous structural and functional damage occurs despite IOP levels within the statistically normal range (6, 7).

Growing evidence suggests that the consequences of glaucoma are not confined to the eye. The retina and optic nerve are extensions of the central nervous system, and glaucoma shares biological features with neurodegenerative disease, including mitochondrial dysfunction, oxidative stress, neuroinflammation, impaired perfusion, and trans-synaptic degeneration (3–5). Recent clinical and epidemiological studies have also linked glaucoma with cognitive impairment, reinforcing the clinical relevance of cognitive status in long-term glaucoma care (8–10).

Recent cohort studies and meta-analyses have linked glaucoma with cognitive impairment and dementia (10–12). However, whether cognitive vulnerability differs between NTG and high-tension glaucoma (HTG) remains uncertain (8). Mechanistically, HTG may be more closely linked to pressure-related optic nerve stress, whereas NTG may reflect a stronger contribution of vascular dysregulation, systemic hypotension, and impaired ocular perfusion (13, 14). Sleep disturbance and psychological symptoms are also frequent in patients with chronic visual disorders and may influence cognition and glaucoma outcomes (15, 16). A recent study integrating visual function, cognitive performance, and transcriptomic profiling further illustrates potential systemic and molecular dimensions of glaucomatous neurodegeneration, although such evidence does not establish a direct causal pathway between glaucoma and cognitive decline (37).

This study aimed to compare the frequency and profile of MoCA-screen-positive status between NTG and HTG in a Chinese POAG cohort and to test whether the main association was robust to visual-field severity, RNFL thickness, vascular and metabolic covariates, and a vision-reduced cognitive score. Subtype-specific clinical, sleep, and ocular associations were prespecified as exploratory. The analysis was designed to evaluate the association in a middle-aged Chinese clinical cohort while addressing visual and systemic confounding. Limited color Doppler imaging observations were retained only as descriptive context and were not used to define a vascular subgroup or construct a prediction model.

## 2 Materials and methods

### 2.1 Study design and participants

This single-center observational study analyzed patients with POAG evaluated at Beijing Tongren Hospital between January 2024 and January 2025. Patients were identified consecutively during the study period. Targeted text-message invitations describing the study purpose, procedures, possible risks, and participant rights were sent to all 143 patients in the registered target population. Ninety-six patients responded and agreed to participate (response rate, 67.1%). Three could not complete the study procedures because of scheduling or geographic constraints, leaving 93 participants for analysis (49 NTG and 44 HTG). Individual-level clinical information was not available for the 47 nonparticipants, so participant and nonparticipant characteristics could not be compared. Participant flow and subtype-stratified MoCA screening status are shown in Figure 1.

**Figure 1:**
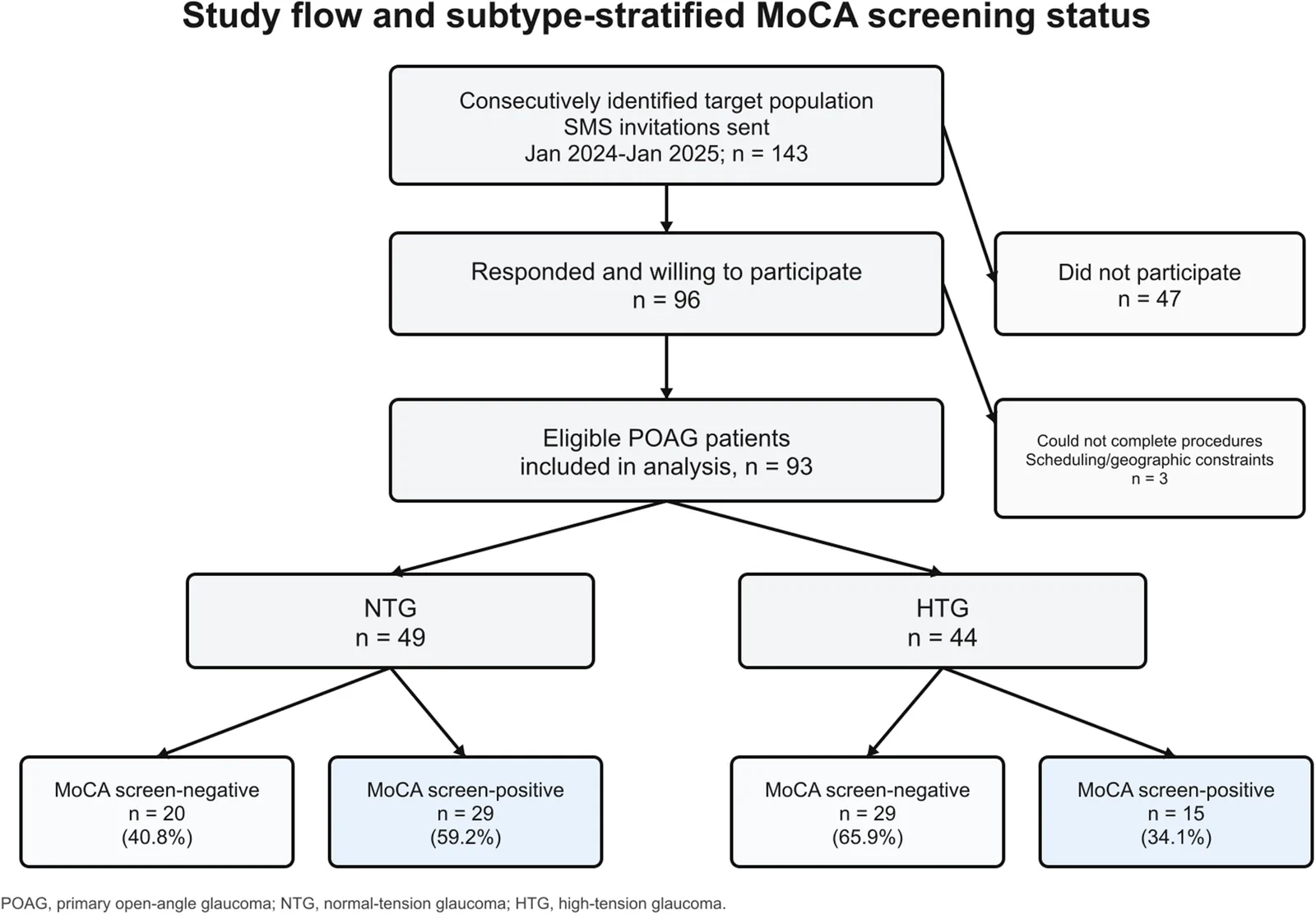
Study flow and subtype-stratified MoCA screening status. All 143 consecutively identified patients in the registered target population received a text-message invitation; 96 responded and agreed to participate. Three could not complete the study procedures because of scheduling or geographic constraints, leaving 93 participants for analysis. Labels denote MoCA screen-positive or screen-negative status and do not constitute a clinical diagnosis of MCI.

The study protocol was approved by the Ethics Committee of Beijing Tongren Hospital, Capital Medical University (approval number: TREC2025-KY064). Written informed consent was obtained from all participants before study-specific cognitive and psychological assessments.

Eligible participants were 40–65 years of age, had open angles on gonioscopy, decimal best-corrected or uncorrected visual acuity greater than 0.6, and were able to complete the required ophthalmic and neuropsychological assessments. Primary open-angle glaucoma was diagnosed by an open anterior-chamber angle, glaucomatous optic nerve head and/or RNFL damage, and corresponding reproducible visual-field defects, after exclusion of secondary glaucoma. In accordance with Chinese guideline and consensus definitions, subtype classification was based on repeated noncontact tonometry (NCT) measurements obtained on at least three separate dates and reviewed with the complete clinical record (17, 18). HTG was defined by an IOP >21 mmHg before treatment or by a documented value >21 mmHg despite ongoing pressure-lowering therapy. NTG required IOP values <=21 mmHg on all three or more dates while untreated or after an appropriate medication washout. Corvis ST assessment, including central corneal thickness and biomechanically corrected IOP, was available for all participants and was considered when checking for possible cornea-related misclassification. A stricter sensitivity analysis retained only HTG participants whose recorded peak IOP was >=25 mmHg.

Patients were excluded if they had previous ocular trauma, tumor, inflammation, laser or incisional ocular surgery, another retinal or optic nerve disease causing visual-field loss, diagnosed psychiatric or central nervous system disease including dementia, severe systemic disease, recent acute infection, inability to cooperate with examinations, or refusal of informed consent.

### 2.2 Clinical and ophthalmic assessment

Demographic information, medical history, smoking and alcohol history, glaucoma duration, family history, and medication use were collected through face-to-face interview and medical-record review. Resting upper-arm blood pressure and pulse were measured in the seated position after at least 5 min of rest, and repeated measurements were averaged. Conventional study-visit seated hypotension was defined as systolic blood pressure <90 mmHg and/or diastolic blood pressure <60 mmHg (38). For the separate exploratory NTG variable, study-defined history of low blood pressure was defined as systolic blood pressure <110 mmHg and/or diastolic blood pressure <65 mmHg on at least three resting seated upper-arm blood-pressure measurements obtained on separate dates, as identified from participant-reported medical history and/or verifiable medical records. Lee et al. reported decision-tree cut points of minimum systolic blood pressure of 108 mmHg for structural progression and minimum diastolic blood pressure of 63 mmHg for progressive macular ganglion cell-inner plexiform layer thinning in treated NTG (39). We therefore used the rounded <110/<65 mmHg operational threshold to represent the same low-blood-pressure range; it was not considered a universal clinical definition or a cognitive-risk threshold. All 49 NTG participants had sufficient historical or medical-record information for assessment under the three-measurement criterion. Twenty-seven participants also underwent repeated multi-time-point upper-arm blood-pressure measurement alongside 24 h IOP monitoring. These measurements were used only as contextual or corroborative information and did not determine classification. Among the 21 participants positive for study-defined history of low blood pressure, 13 had also undergone multi-time-point measurement and eight had not; no participant was classified as positive solely on the basis of a single multi-time-point measurement. This variable was ascertained only in NTG participants and was included only in the exploratory NTG Firth model; equivalent history data were not collected for HTG participants, and a subtype interaction was therefore not estimable. Orthostatic testing and formal 24 h ambulatory blood-pressure monitoring, including standardized nocturnal dipping assessment, were not performed systematically (14, 19).

Ophthalmic examinations included uncorrected visual acuity, best-corrected visual acuity (BCVA, decimal notation), slit-lamp examination, gonioscopy, noncontact tonometry using the Canon TX-20 (Canon Inc., Tokyo, Japan), and Corvis ST assessment (Oculus, Wetzlar, Germany), including central corneal thickness (CCT) and biomechanically corrected IOP (bIOP). Other examinations included optic disc assessment, standard automated perimetry using the Humphrey Field Analyzer 24-2 program (Carl Zeiss Meditec, Dublin, CA, USA), and optical coherence tomography. Visual-field indices included VFI, mean deviation (MD), and pattern standard deviation (PSD). Peripapillary RNFL thickness was measured using Cirrus HD-OCT (Carl Zeiss Meditec, Dublin, CA, USA) with the Optic Disc Cube 200 × 200 protocol. Because cognition was analyzed at the participant level, bilateral means were used for BCVA, current IOP, CCT, RNFL, VFI, MD, and PSD.

In a subset of 25 participants with NTG who had complete color Doppler imaging measurements, peak systolic velocity, end-diastolic velocity, and resistive index of the central retinal artery and posterior ciliary artery were summarized using a worse-eye strategy and reviewed descriptively alongside systemic blood pressure and cognitive findings. Given the small sample size and the absence of a same-center healthy reference group, these measurements were treated only as exploratory contextual information and were not used to establish normative reference limits, define blood-flow abnormality, construct a composite blood-flow index, or develop an independent model (13).

### 2.3 Cognitive, psychological and sleep assessment

Cognitive function was screened using version 7.1 of the Chinese Montreal Cognitive Assessment (MoCA), a 30-point instrument covering multiple cognitive domains (20, 40). Following the standard scoring rule, one point was added for participants with <=12 years of education, up to a maximum score of 30. A corrected score <26 was classified as MoCA screen-positive; this was a screening definition rather than a clinical diagnosis of mild cognitive impairment. Because eight MoCA points depend substantially on vision, a vision-reduced MoCA-22 score was calculated as corrected total MoCA minus the visuospatial/executive (5 points) and naming (3 points) domains. Continuous MoCA-22 was the primary visual-confounding sensitivity outcome; MoCA-22 <18 was examined only as an exploratory threshold and was not substituted for the prespecified primary endpoint (20–22).

Anxiety was assessed with the Hamilton Anxiety Rating Scale (HAMA), depressive symptoms with the 24-item Hamilton Depression Rating Scale (HAMD), and sleep quality with the Pittsburgh Sleep Quality Index (PSQI). Two trained assessors conducted the interviews and scoring according to standardized procedures (15, 23, 24).

### 2.4 Statistical analysis

Continuous variables are presented as mean (standard deviation) or median [interquartile range], according to distribution; categorical variables are counts and percentages. Between-group comparisons used Welch *t* tests or two-sided Mann–Whitney *U* tests for continuous variables and chi-square, Fisher exact, or exact fixed-margin permutation chi-square tests for categorical variables, as appropriate. Benjamini–Hochberg false-discovery-rate correction was applied separately to MoCA total/domain comparisons and to exploratory psychological/sleep comparisons within each subtype. The primary binary comparison used conventional logistic regression in six sequential models: crude (Model 0); age, sex, and education (Model 1); plus VFI (Model 2); plus RNFL (Model 3); plus diabetes (Model 4); and plus systolic blood pressure (Model 5). Sensitivity analyses replaced VFI with MD, added BCVA or CCT, and excluded HTG cases with peak historical IOP <25 mmHg. Continuous corrected MoCA and MoCA-22 were analyzed with linear models using HC3 robust standard errors. Subtype-by-PSQI and subtype-by-RNFL interactions were tested in common-covariate logistic models, with Firth penalized sensitivity analyses; comparable subtype-specific Firth models included age, sex, education, PSQI, and RNFL. A subtype interaction for study-defined history of low blood pressure was not estimated because equivalent ascertainment was unavailable in HTG participants. Spearman correlations assessed RNFL with VFI, MD, and PSD, with within-stratum FDR correction. The NTG PSQI coefficient was re-estimated after adding HAMA, HAMD, and both mood scores. RNFL effects were additionally reported per 5 micrometers thinner. Apparent AUC 95% CIs used 5,000 nonparametric bootstrap resamples; calibration intercept and slope CIs used 2,000 resamples; and optimism-corrected AUC and Brier scores used 1,000 resamples. DeLong tests compared apparent in-sample AUCs and were Holm-adjusted within subtype. All performance analyses represent internal assessment rather than external validation. Tests were two-sided with *P* < 0.05 or *q* < 0.05. Statistical analyses were performed using Python 3.13 with SciPy 1.17.0, statsmodels 0.14.6, and scikit-learn 1.8.0.

### 2.5 Use of generative artificial intelligencey

ChatGPT/Codex (OpenAI, San Francisco, CA, USA) was used only to assist with English-language editing and wording refinement. It was not used to generate original data, perform statistical analyses, interpret results, or draw scientific conclusions. All AI-assisted text was reviewed, verified, and edited by the authors, who retain full responsibility for the accuracy and integrity of the manuscript.

## 3 Results

### 3.1 Baseline characteristics

Baseline characteristics are summarized in Table 1. HTG patients had higher BMI (*P* = 0.002), systolic blood pressure (*P* < 0.001), diastolic blood pressure (*P* = 0.023), pulse rate (*P* = 0.043), current IOP (*P* < 0.001), peak historical IOP used for classification [27.0 (25.8, 30.0) vs. 15.0 (13.8, 17.0) mmHg; *P* < 0.001], CCT (*P* = 0.023), and VFI (*P* = 0.026). Conventional study-visit seated hypotension occurred in 6/49 NTG and 1/44 HTG participants (Fisher exact *P* = 0.115). Age, sex, education distribution, glaucoma duration, medication number, RNFL thickness, MD, and PSD did not differ significantly between subtypes.

**Table 1:** Baseline characteristics of NTG and HTG participants.

Variable	NTG (*n* = 49)	HTG (*n* = 44)	Test statistic	*P* value
Age, years	52.0 [47.0, 60.0]	50.0 [47.0, 57.0]	*U* = 1,219.0	0.279
Sex (male), *n* (%)	23 (46.9%)	26 (59.1%)	chi-square = 1.37	0.241
Education level, *n* (%)			chi-square = 3.50	0.174
< = High school	7 (14.3%)	13 (29.5%)		
College/undergraduate	23 (46.9%)	19 (43.2%)		
Postgraduate	19 (38.8%)	12 (27.3%)		
BMI, kg/m^2^	22.40 [20.30, 24.80]	23.80 [22.68, 25.85]	*U* = 667.0	0.002
SBP, mmHg	114.18 ± 17.78	125.45 ± 10.84	*t* = −3.73	<0.001
DBP, mmHg	75.00 [62.00, 84.00]	80.00 [73.75, 87.25]	*U* = 781.5	0.023
Pulse, beats/min	69.20 ± 10.16	73.55 ± 10.22	*t* = −2.05	0.043
Glaucoma duration, years	1.00 [0.42, 4.00]	0.96 [0.33, 1.50]	*U* = 1,253.0	0.178
Number of current medications	1.00 [1.00, 2.00]	1.00 [1.00, 2.00]	*U* = 944.0	0.209
Hypertension, *n* (%)	11 (22.4%)	15 (34.1%)	chi-square = 1.56	0.212
Diabetes mellitus, *n* (%)	11 (22.4%)	5 (11.4%)	chi-square = 2.00	0.157
Hyperlipidaemia, *n* (%)	27 (55.1%)	17 (38.6%)	chi-square = 2.52	0.112
Smoking history, *n* (%)	12 (24.5%)	12 (27.3%)	chi-square = 0.09	0.759
Alcohol history, *n* (%)	18 (36.7%)	13 (29.5%)	chi-square = 0.54	0.463
Family history of glaucoma, *n* (%)	11 (22.4%)	9 (20.5%)	chi-square = 0.05	0.815
Mean BCVA (decimal)	1.00 [1.00, 1.10]	1.00 [0.90, 1.00]	*U* = 1,148.5	0.568
Current mean IOP, mmHg	13.00 [12.25, 14.55]	19.27 [17.84, 21.50]	*U* = 159.5	<0.001
Peak historical IOP used for classification, mmHg	15.00 [13.80, 17.00]	27.00 [25.80, 30.00]	*U* = 0.0	<0.001
Conventional study-visit seated hypotension, *n* (%)	6 (12.2%)	1 (2.3%)	OR = 6.00	0.115
Mean CCT, micrometers	532.50 [502.50, 546.00]	547.50 [518.62, 571.50]	*U* = 782.0	0.023
Mean RNFL thickness, micrometers	71.50 [70.00, 75.00]; range 62.0–82.0	73.75 [66.00, 83.88]; range 55.5–101.5	*U* = 979.0	0.448
Mean VFI, %	82.50 [74.00, 89.50]	91.25 [74.88, 95.38]	*U* = 789.0	0.026
Mean MD, dB	−6.14 [−8.63, −4.17]	−4.70 [−9.40, −2.65]	*U* = 969.5	0.406
Mean PSD, dB	7.28 ± 2.67	6.43 ± 3.31	*t* = 1.35	0.181

Data are mean ± SD, median [IQR], or *n* (%). Ophthalmic variables are bilateral means. BCVA is decimal. Peak IOP is the historical value used for subtype classification; HTG values could be recorded before treatment or despite ongoing therapy. Conventional study-visit seated hypotension is SBP <90 mmHg and/or DBP <60 mmHg. *t*, *U*, chi-square, and OR denote Welch t, Mann–Whitney *U*, chi-square, and Fisher exact odds-ratio statistics, respectively. Statistically significant between-group differences at *P* < 0.05 are shown in bold; these comparisons are descriptive and unadjusted.

### 3.2 MoCA screening status and domain scores

Across all POAG patients, 44 of 93 participants were MoCA screen-positive (47.3%). The frequency was higher in NTG than HTG (29/49, 59.2% vs. 15/44, 34.1%; continuity-corrected chi-square *P* = 0.027; Table 2), corresponding to a crude OR of 2.80 (95% CI: 1.20–6.52; Wald *P* = 0.017). Across sequential models, the NTG estimate was 8.69 after demographic adjustment, 8.05 after adding VFI, 7.10 after RNFL, 7.50 after diabetes, and 8.47 (95% CI: 1.81–39.61; *P* = 0.007) after systolic blood pressure (Table 3).

**Table 2:** MoCA screening status and corrected MoCA scores by POAG subtype.

Measure	NTG (*n* = 49)	HTG (*n* = 44)	Statistic	Raw P	FDR q/note
MoCA screen-positive, *n* (%)	29 (59.2%)	15 (34.1%)	chi-square = 4.89	0.027	Primary outcome
Total corrected MoCA	25.0 [23.0, 27.0]	27.0 [24.0, 27.0]	*U* = 791.0	0.026	0.106
Visuospatial/executive	5.0 [4.0, 5.0]	5.0 [4.0, 5.0]	*U* = 1,131.5	0.630	0.720
Naming	3.0 [2.0, 3.0]	3.0 [3.0, 3.0]	*U* = 875.5	0.029	0.106
Attention	6.0 [5.0, 6.0]	6.0 [6.0, 6.0]	*U* = 883.0	0.040	0.106
Language	2.0 [1.0, 2.0]	2.0 [1.0, 2.0]	*U* = 900.5	0.134	0.267
Abstraction	2.0 [2.0, 2.0]	2.0 [2.0, 2.0]	*U* = 1,022.5	0.409	0.545
Delayed recall	3.0 [2.0, 4.0]	3.0 [2.0, 3.2]	*U* = 1,051.5	0.837	0.837
Orientation	6.0 [6.0, 6.0]	6.0 [6.0, 6.0]	*U* = 1,056.0	0.354	0.545

MoCA values are median [IQR]. MoCA <26 denotes screen-positive status, not a clinical diagnosis. The primary status outcome is not FDR-adjusted; *q* values use Benjamini–Hochberg correction for total/domain scores. All Mann–Whitney tests are two-sided. The attention-domain comparison yielded *P* = 0.040; FDR-adjusted q values for total MoCA, naming, and attention were all 0.106. No total- or domain-score comparison remained significant after FDR correction.

**Table 3:** Sequential models for NTG vs. HTG and MoCA-screen-positive status.

Model	Adjustment set	NTG vs. HTG OR (95% CI)	*P* value
Model 0	Crude	2.80 (1.20–6.52)	0.017
Model 1	Age + sex + education	8.69 (2.41–31.37)	0.001
Model 2	Model 1 + VFI	8.05 (2.19–29.61)	0.002
Model 3	Model 2 + RNFL	7.10 (1.75–28.85)	0.006
Model 4	Model 3 + diabetes	7.50 (1.69–33.32)	0.008
Model 5	Model 4 + systolic BP	8.47 (1.81–39.61)	0.007

Conventional logistic regression; all models use *n* = 93. Model 0 is crude; Model 1 adds age, sex, and education; Model 2 adds VFI; Model 3 adds RNFL; Model 4 adds diabetes; Model 5 adds systolic blood pressure. OR >1 indicates higher odds in NTG. These are explanatory confounding models, not externally validated prediction models.

Vision-reduced MoCA-22 was lower in NTG than HTG [18 (16, 19) vs. 19 (17.75, 20); *P* = 0.043]. After adjustment for age, sex, education, VFI, and RNFL, NTG was associated with a 0.82-point lower MoCA-22 score (95% CI: −1.53 to −0.11; *P* = 0.024); the estimate was −0.88 (95% CI: −1.63 to −0.14; *P* = 0.020) after additionally adjusting for diabetes and systolic blood pressure. The fully adjusted continuous corrected-MoCA estimate was −1.07 (95% CI: −1.98 to −0.16; *P* = 0.021). The exploratory MoCA-22 <18 analysis yielded an adjusted OR of 3.94 (95% CI: 1.24–12.49; *P* = 0.020; Supplementary Table S2). The two-sided attention-domain comparison yielded *P* = 0.040. After FDR correction, total MoCA, naming, and attention each had *q* = 0.106; no total- or domain-score comparison remained significant (Table 2).

### 3.3 Subtype-specific associations of anxiety, depression, and sleep quality with MoCA-screen-positive status

Anxiety showed exploratory within-subtype associations with MoCA screening status. In NTG, anxiety severity distribution (raw *P* = 0.002; *q* = 0.009) and dichotomized anxiety status (raw *P* = 0.004; *q* = 0.012; AUC = 0.712) remained significant after FDR correction. In HTG, anxiety severity distribution remained significant (raw *P* = 0.010; *q* = 0.039), whereas somatic anxiety and dichotomized anxiety status were borderline after correction (both *q* = 0.052). These unadjusted findings are reported as exploratory in Supplementary Table S1.

Depressive symptoms showed no consistent association with MoCA screening status. HAMD total score, depression severity distribution, and dichotomized depression status were not significant after FDR correction in either subtype (Supplementary Table S1).

Sleep quality showed the most consistent non-ophthalmic signal. PSQI total score was higher in MoCA screen-positive participants in both NTG (raw *P* < 0.001; *q* < 0.001; AUC = 0.856) and HTG (raw *P* = 0.002; *q* = 0.014; AUC = 0.790).

### 3.4 Adjusted and subtype-specific multivariable models

In the NTG subgroup, 29 of 49 participants were MoCA screen-positive. All 49 NTG participants were assessable under the historical or medical-record criterion for study-defined history of low blood pressure. Twenty-seven also underwent supplementary multi-time-point blood-pressure measurement alongside 24 h IOP monitoring. All 21 history-positive participants met the requirement for at least three qualifying measurements on separate dates; 13 of these 21 had also undergone multi-time-point measurement and eight had not. Study-defined history of low blood pressure was present in 17/29 screen-positive and 4/20 screen-negative participants (crude OR: 5.67; Fisher *P* = 0.009). In an exploratory Firth model, study-defined history of low blood pressure (OR: 10.57, 95% CI: 1.35–82.97; *P* = 0.025), higher PSQI score (OR: 1.43 per point, 95% CI: 1.03–1.98; *P* = 0.031), and thinner RNFL (OR: 5.86 per 5 micrometers thinner, 95% CI: 1.43–24.08; *P* = 0.014) were associated with screen-positive status (Table 4). The wide intervals and limited event count indicate substantial imprecision. Limited CDI observations were not included in inferential analyses.

**Table 4:** Exploratory subtype-specific firth penalized logistic regression models.

Subtype	Predictor	OR (95% CI)	*P* value
NTG	Age, per year	0.99 (0.87–1.13)	0.900
NTG	Education, per category	0.46 (0.12–1.79)	0.262
NTG	Study-defined history of low blood pressure	10.57 (1.35–82.97)	0.025
NTG	PSQI, per point	1.43 (1.03–1.98)	0.031
NTG	RNFL, per 5 micrometers thinner	5.86 (1.43–24.08)	0.014
HTG	Age, per year	1.12 (0.98–1.28)	0.090
HTG	Anxiety status	1.45 (0.21–9.80)	0.706
HTG	PSQI, per point	1.22 (0.93–1.59)	0.148
HTG	RNFL, per 5 micrometers thinner	1.55 (1.01–2.38)	0.043

Firth penalized logistic regression was used because subtype event counts were limited. Study-defined history of low blood pressure was ascertained only in NTG participants and was included only in the exploratory NTG model; equivalent data were not collected for HTG. Positivity required at least three qualifying resting seated upper-arm measurements on separate dates at systolic blood pressure <110 mmHg and/or diastolic blood pressure <65 mmHg. All 49 NTG participants were assessed under this criterion; 27 also had supplementary multi-time-point measurements, which did not determine classification. RNFL is reported per 5 micrometers thinner. Wide CIs indicate substantial imprecision; models are exploratory.

In the HTG subgroup, 15 of 44 participants were MoCA screen-positive. In the exploratory Firth model, thinner RNFL was associated with screen-positive status (OR: 1.55 per 5 micrometers thinner, 95% CI: 1.01–2.38; *P* = 0.043). Anxiety status (*P* = 0.706) and PSQI score (*P* = 0.148) were not independently associated (Table 4). These within-subtype significance patterns do not by themselves demonstrate effect differences between NTG and HTG.

Internal model performance is summarized in Table 5. Apparent AUCs were 0.881 (95% CI: 0.806–0.943) overall, 0.940 (0.869–0.988) for NTG, and 0.892 (0.763–0.989) for HTG; bootstrap-corrected AUCs were 0.853, 0.900, and 0.851. Corrected Brier scores were 0.167, 0.140, and 0.155. Calibration intercepts/slopes were 0.00/0.94 overall, 0.03/1.15 in NTG, and −0.01/1.10 in HTG, with wide bootstrap CIs. DeLong comparisons used apparent in-sample AUCs (Table 6). The NTG combined model did not outperform PSQI alone (Holm *P* = 0.120), and the HTG combined model did not outperform RNFL alone (*P* = 0.076). ROC curves are shown in Figure 2; adjusted estimates are shown in Figure 3; apparent calibration plots are provided in Supplementary Figure S1.

**Table 5:** Apparent discrimination, calibration, and bootstrap internal validation.

Model	Apparent AUC (95% CI)	Corrected AUC	Apparent brier	Corrected brier	Calibration intercept (95% CI)	Calibration slope (95% CI)
Overall	0.881 (0.806–0.943)	0.853	0.142	0.167	0.00 (−0.60–0.56)	0.94 (0.66–1.57)
NTG	0.940 (0.869–0.988)	0.900	0.099	0.140	0.03 (−1.05–1.00)	1.15 (0.81–2.19)
HTG	0.892 (0.763–0.989)	0.851	0.120	0.155	−0.01 (−0.85–0.83)	1.10 (0.64–2.61)

Apparent AUC CIs use 5,000 nonparametric bootstrap resamples; calibration CIs use 2,000 resamples; optimism correction uses 1,000 resamples with locked seed 20,26,0,818. Corrected estimates provide internal validation only and do not establish performance in an independent cohort.

**Table 6:** Delong comparisons of apparent in-sample AUCs.

Subtype	Comparison	Model AUC	Comparator AUC	*Z*	Raw *P*	Holm *P*
NTG	Combined vs. study-defined low-BP history	0.940	0.693	3.747	<0.001	<0.001
NTG	Combined vs. PSQI	0.940	0.856	1.555	0.120	0.120
NTG	Combined vs. RNFL	0.940	0.797	2.554	0.011	0.021
HTG	Combined vs. RNFL	0.892	0.767	1.776	0.076	0.076
HTG	Combined vs. anxiety status	0.892	0.694	3.116	0.002	0.004

Paired ROC curves were compared using DeLong tests with Holm adjustment within subtype. All AUCs in this table are apparent in-sample values; bootstrap-corrected AUCs are not used in DeLong tests. For RNFL, lower thickness was coded as risk.

**Figure 2:**
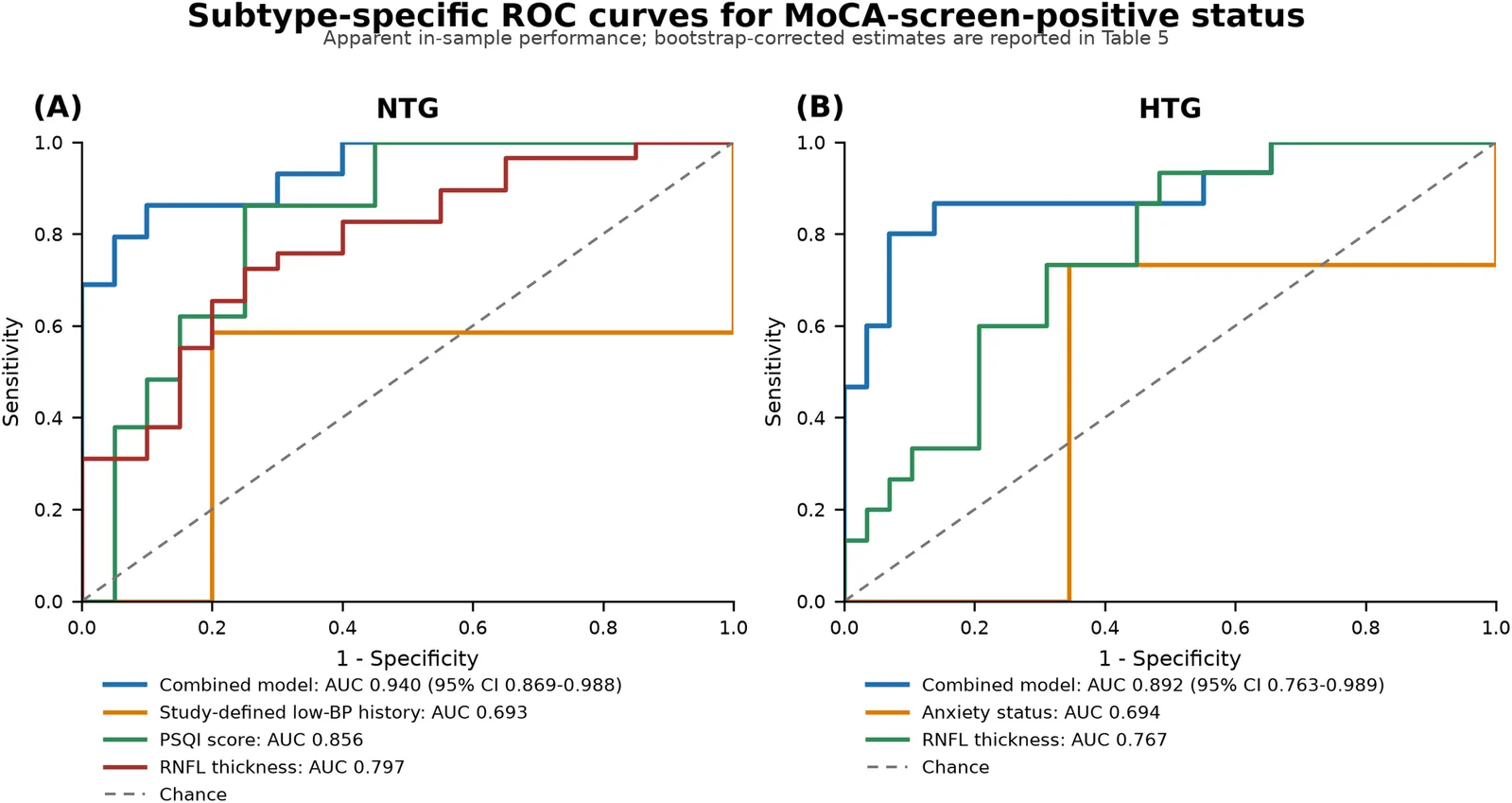
Subtype-specific receiver operating characteristic curves for MoCA-screen-positive status. **(A)** NTG Firth model and individual comparators. **(B)** HTG Firth model and individual comparators. Combined-model AUCs are apparent in-sample estimates with bootstrap 95% CIs. DeLong tests compare apparent paired curves; optimism-corrected estimates are reported in Table 5. The history-of-hypotension comparator in panel **A** is the study-defined NTG-only variable. The low-blood-pressure comparator in panel **A** is the NTG-only study-defined history variable.

**Figure 3:**
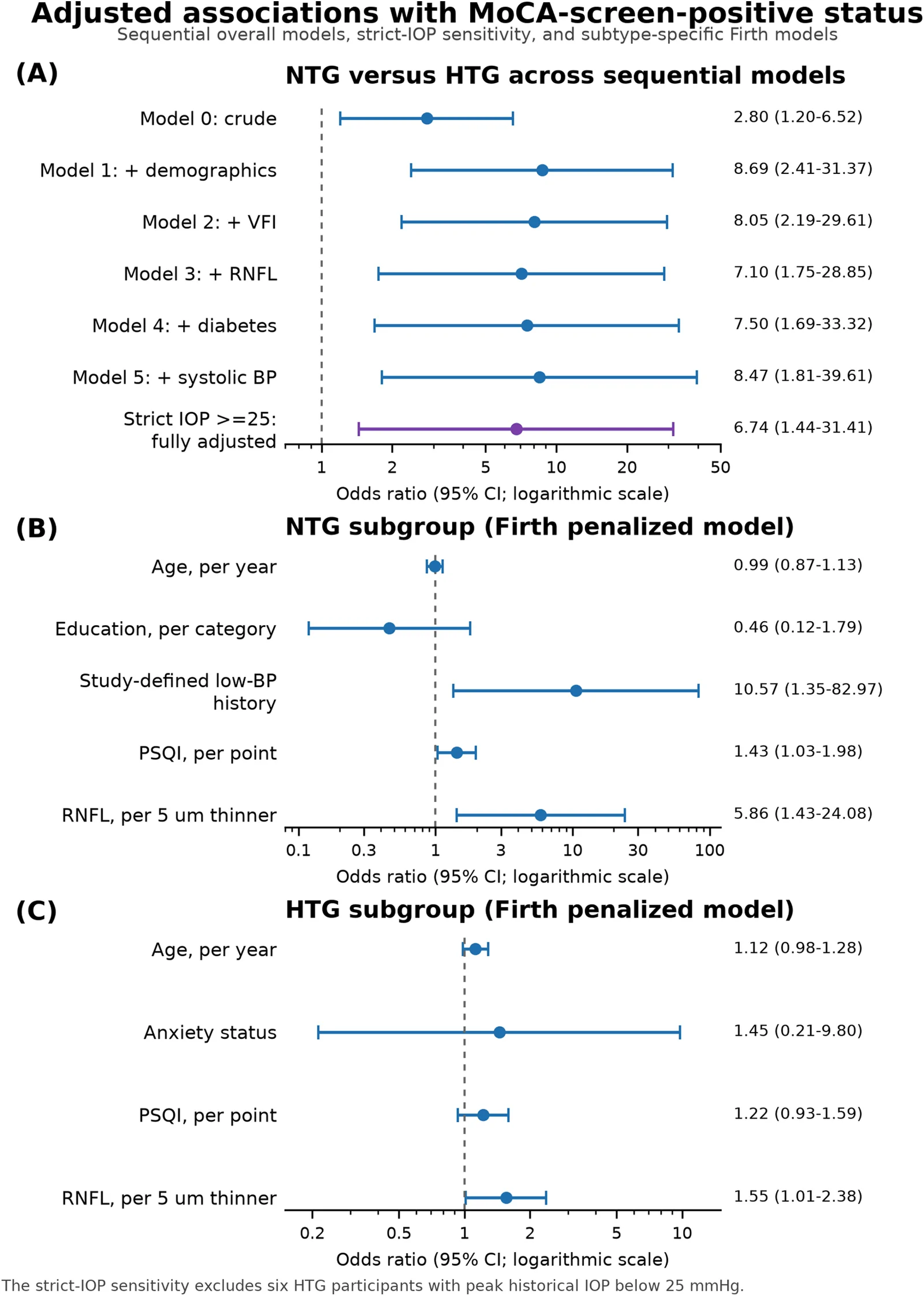
Adjusted odds ratios for MoCA-screen-positive status. **(A)** NTG-vs.-HTG estimates across Models 0–5 and the fully adjusted strict-IOP sensitivity analysis. **(B)** Exploratory NTG Firth model. **(C)** Exploratory HTG Firth model. RNFL effects are reported per 5 micrometers thinner. Error bars indicate 95% CIs. In panel B, history of hypotension refers to the study-defined variable ascertained only in NTG participants. In panel B, the low-blood-pressure term is the NTG-only study-defined history variable.

### 3.5 Sensitivity, interaction, and structure-function analyses

Formal subtype interactions were not significant for PSQI (interaction OR: 1.11, 95% CI: 0.74–1.68; *P* = 0.604; Firth sensitivity *P* = 0.612) or RNFL (interaction OR: 0.77, 95% CI: 0.58–1.03; *P* = 0.082; Firth sensitivity *P* = 0.092). In common-covariate Firth models including age, sex, education, PSQI, and RNFL, the PSQI/RNFL estimates were 1.32/0.77 in NTG and 1.18/0.95 in HTG. A subtype interaction for study-defined history of low blood pressure was not estimable because equivalent history data were not collected for HTG participants (Supplementary Table S3).

Six HTG participants had peak historical IOP between 22 and 24.8 mmHg. Excluding them left 38 HTG participants; the fully adjusted NTG-vs.-HTG association remained (OR: 6.74, 95% CI: 1.44–31.41; *P* = 0.015). Results were also directionally stable when VFI was replaced by MD and when BCVA or CCT was added. In NTG, the PSQI estimate remained similar after HAMA adjustment (OR: 1.39), HAMD adjustment (OR: 1.46), and joint adjustment (OR: 1.47; all *P* <= 0.048; Supplementary Table S5).

In the overall cohort, RNFL correlated with VFI (Spearman rho = 0.482), MD (rho = 0.419), and PSD (rho = −0.495; all *q* < 0.001). Correlations were strong in HTG (absolute rho 0.607–0.732; all *q* < 0.001) but not significant in NTG after correction (Supplementary Table S4). These subtype differences are descriptive and may reflect sample size, range, or disease-pattern differences rather than established effect modification.

## 4 Discussion

In this single-center POAG cohort, MoCA-screen-positive status was more frequent in NTG than HTG. The association persisted across sequential adjustment for demographics, VFI, RNFL, diabetes, and systolic blood pressure, in a strict peak-IOP sensitivity analysis, and when cognition was analyzed with a vision-reduced continuous MoCA-22 score. These findings support a robust subtype association within the measured covariates. However, the PSQI and RNFL interaction tests were not significant, and study-defined history of low blood pressure could not be compared across subtypes. The data therefore do not establish distinct biological NTG and HTG phenotypes.

Previous studies have increasingly linked glaucoma with cognitive impairment and dementia, but few have compared IOP-defined subtypes. Mullany et al. reported a stronger association of NTG with cognitive impairment in adults aged >=65 years using a 22-point vision-independent assessment and stricter pressure separation (8). Our cohort included adults aged 40–65 years, used a Chinese-language standard MoCA with a derived MoCA-22 sensitivity score, and defined HTG at >21 mmHg under Chinese guidance. These demographic and methodological differences limit direct comparability, while the consistent direction of association supports further investigation of cognitive differences across IOP-defined POAG subtypes.

At the domain level, NTG participants had nominally lower naming and attention scores, alongside a lower corrected total MoCA score; however, none of these comparisons remained significant after FDR correction (all *q* = 0.106). Visuospatial/executive, language, abstraction, delayed recall, and orientation scores did not show corrected between-group differences. The persistence of the group difference in the vision-reduced MoCA-22, which omits visuospatial/executive and naming items, argues against a purely vision-dependent explanation but does not identify a discrete cognitive phenotype. Glaucoma-related retinal and central nervous system changes have been linked to disturbances across visual, attentional, memory, emotional, and other higher-order networks (41). Nevertheless, brief MoCA domains have restricted score ranges and are not equivalent to formal neuropsychological testing. The naming and attention pattern should therefore be regarded as hypothesis-generating rather than evidence of selective neuronal or neurodegenerative damage.

RNFL thinning was associated with MoCA screening status in the fitted models and correlated with VFI, MD, and PSD in the overall cohort. This may reflect glaucoma severity, parallel retinal and cognitive change, visual-function effects on testing, or a shared neurodegenerative process. The literature is mixed: OCT meta-analyses and population studies support associations between retinal neuronal layers and cognition (25–30), whereas Bastani Viarsagh et al. did not identify a significant RNFL-MoCA association in glaucoma (9), and Eppenberger et al. identified GCIPL rather than RNFL as the more sensitive longitudinal marker (31). Moreover, RNFL did not differ significantly between NTG and HTG in our cohort, while VFI did. RNFL should therefore be described as an ocular structural correlate, not as evidence of a shared causal neurodegenerative pathway.

A recent middle-aged population cohort reported associations of thinner RNFL and ganglion cell layer with cognitive performance and of thinner RNFL with decline in processing speed from childhood to adulthood (42). That study also emphasized the need for further longitudinal work to establish temporal order and address confounding. It supports the plausibility of a retinal-cognitive association, but not a causal or glaucoma-specific interpretation of the RNFL finding in the present cross-sectional cohort.

PSQI was an exploratory correlate in NTG, and its coefficient remained similar after separate and joint adjustment for HAMA and HAMD. However, the subtype-by-PSQI interaction was not significant, and poor sleep may reflect mood, visual impairment, medication burden, glaucoma severity, reverse causation, or other unmeasured factors. Prior glaucoma studies have reported circadian and sleep-quality disturbances (32, 33), but the present cross-sectional data cannot determine whether sleep disturbance precedes, follows, or shares causes with lower cognitive performance.

The association of study-defined history of low blood pressure with MoCA-screen-positive status in NTG was based on 21 history-positive participants and had a very wide confidence interval. Every positive classification required at least three qualifying resting seated upper-arm measurements on separate dates from the reported history or available medical records; the multi-time-point series obtained alongside 24 h IOP monitoring was supplementary and was not an alternative single-measurement route. Lee et al. reported decision-tree cut points of minimum systolic blood pressure of 108 mmHg for structural progression and minimum diastolic blood pressure of 63 mmHg for progressive macular ganglion cell-inner plexiform layer thinning in treated NTG (39). Our <110/<65 mmHg threshold was a pragmatic rounding of this low-blood-pressure range and should not be interpreted as a universal diagnostic threshold or a validated cognitive-risk cutoff. Charlson et al. examined nocturnal hypotension in relation to visual-field progression, not cognition (34). Because the history variable was ascertained only in NTG participants, differential ascertainment between subtypes cannot be excluded. Within NTG, reliance on reported history or clinical records and the nonuniform availability of supplementary multi-time-point measurements may also permit residual measurement or classification error. Availability of the supplementary assessment was not formally compared by glaucoma severity or MoCA-screening status because it did not determine classification and the assessed subset was limited. The association therefore remains an exploratory within-NTG finding that motivates, but does not confirm, a perfusion-related hypothesis.

In HTG, RNFL thickness was the only independent correlate in the exploratory penalized model, while anxiety severity was an unadjusted signal. Psychological symptoms are common in glaucoma (23, 35, 36), but the current results do not identify anxiety as an independent HTG mechanism. Common-covariate subtype models and formal interactions likewise did not demonstrate statistically distinct PSQI or RNFL effects. Differences in within-subtype *P* values should not be interpreted as differences between subtypes.

One possible connection among retinal ganglion-cell injury, sleep, and neurobehavioral symptoms may involve intrinsically photosensitive retinal ganglion cells (ipRGCs). These melanopsin-expressing cells contribute to non-image-forming responses, including circadian photoentrainment, pupillary light responses, sleep-alertness regulation, and mood (43, 44). A small clinical study in glaucoma reported reduced ipRGC-mediated pupillary responses and seasonal neurohormonal differences, providing a rationale for further investigation of this pathway (45). In the present study, however, anxiety and RNFL were separate exploratory correlates; no direct anxiety-RNFL relationship was tested, peripapillary RNFL is not an ipRGC-specific measure, and no melanopsin-directed pupillometry, actigraphy, melatonin profiling, or other ipRGC functional measure was obtained. This proposed pathway is therefore an exploratory hypothesis, not an explanation established by our data. Prospective studies combining OCT-derived RNFL/GCIPL measures with chromatic pupillometry, objective circadian and sleep measures, and validated mood and cognitive assessments could test whether ipRGC dysfunction contributes to neurobehavioral vulnerability in glaucoma.

The high apparent AUCs require cautious interpretation. Optimism correction lowered AUCs and worsened Brier scores, calibration intervals were wide, and the combined models did not significantly outperform the strongest single predictor in either subtype. DeLong comparisons used apparent in-sample curves. These analyses describe internal classification in the development sample and should not be described as prediction, risk profiling, or evidence of performance in an independent population.

This study has several limitations. First, it was a single-center, cross-sectional study in a tertiary referral hospital and did not include an age- and education-matched healthy control group. We could therefore compare NTG with HTG, but could not determine whether the overall 47.3% screen-positive proportion exceeded that of the source population. Although patients were identified consecutively and all 143 registered patients were invited, 47 did not participate and their clinical characteristics were unavailable; selection bias and limited generalizability remain possible. Three additional patients could not complete study procedures because of scheduling or geographic constraints. Second, MoCA is a screening tool; although decimal acuity eligibility, VFI adjustment, continuous MoCA, and MoCA-22 analyses reduced visual-confounding concerns, no formal neuropsychological diagnosis or functional assessment was available. Third, subgroup event counts were limited, confidence intervals were wide, interactions were underpowered, and bootstrap correction was internal only. Fourth, subtype classification used at least three NCT measurements and Corvis ST assessment, but standardized diurnal IOP curves were not available. Fifth, study-defined history of low blood pressure was ascertained only in NTG. The same requirement of at least three qualifying measurements on separate dates was applied to all 49 NTG participants, but the supplementary multi-time-point assessment was available in only 27/49 and its availability was not formally examined in relation to glaucoma severity or MoCA-screening status. Patient-reported history and clinical-record ascertainment may introduce recall or measurement error, and equivalent data were unavailable in HTG, so differential ascertainment between subtypes cannot be excluded. Formal ambulatory and orthostatic blood-pressure assessment was not standardized across both subtypes. Sixth, diabetes and other vascular factors may still confound associations despite sensitivity adjustment. Finally, macular GCC/GCIPL and optic nerve head measurements were available only in a subset of patients, while BMO-MRW, OCT angiography, choroidal perfusion, neuroimaging, and molecular biomarkers were not collected systematically. These measures could not be included without introducing substantial missing-data bias.

In conclusion, NTG was associated with more frequent MoCA-screen-positive status and lower vision-reduced MoCA scores than HTG after measured demographic, visual, vascular, and metabolic adjustment. RNFL, sleep, and hypotension findings should be treated as imprecise exploratory correlates rather than evidence of distinct causal phenotypes or clinically validated prediction models. Larger multicenter prospective studies with healthy controls, comprehensive neuropsychological testing, standardized untreated and diurnal IOP ascertainment, ambulatory blood-pressure monitoring, multimodal OCT/OCTA, and independent external validation are needed to clarify temporal and potentially causal relationships.

## Data Availability

The datasets presented in this article are not readily available because; the datasets generated and analyzed during the current study are not publicly available because of participant privacy and institutional data-governance requirements, but may be available from the corresponding author on reasonable request, subject to ethics approval and institutional data-governance requirements. Identifiable participant data will not be shared. Requests to access the datasets should be directed to; Guoping Qing, gptsing@126.com.
