# Supplementary material for: MoCA-screen-positive status differs across intraocular pressure subtypes of primary open-angle glaucoma
Source: Front Med (Lausanne). 2026 Sep 24;13:1912812. doi: 10.3389/fmed.2026.1912812 (PMC13645557; doi:10.3389/fmed.2026.1912812)
Supplement: Supplementary file 1 [file Table1.docx]

**Supplementary Table 1. Exploratory psychological and sleep analyses**

## Supplementary Table 1A. NTG

| **Measure** | **MoCA screen-positive** | **MoCA screen-negative** | **Test** | **Statistic/OR** | **Raw P** | **FDR q** | **AUC** |
| --- | --- | --- | --- | --- | --- | --- | --- |
| HAMA total | 9.0 [5.0, 10.0] | 4.0 [3.0, 10.0] | Mann-Whitney | 361.500 | 0.146 | 0.234 | 0.623 |
| HAMA somatic | 4.0 [2.0, 5.0] | 2.0 [1.0, 3.2] | Mann-Whitney | 365.500 | 0.124 | 0.234 | 0.630 |
| HAMD total | 8.0 [6.0, 13.0] | 6.5 [4.5, 9.2] | Mann-Whitney | 353.500 | 0.198 | 0.265 | 0.609 |
| PSQI total | 8.0 [7.0, 12.0] | 4.0 [4.0, 5.5] | Mann-Whitney | 496.500 | <0.001 | <0.001 | 0.856 |
| Anxiety status | 21/29 (72.4%) | 6/20 (30.0%) | Fisher | 6.125 | 0.004 | 0.012 | 0.712 |
| Depression status | 13/29 (44.8%) | 6/20 (30.0%) | Fisher | 1.896 | 0.377 | 0.431 | 0.574 |
| Anxiety severity | Possible 19; none 8; definite 2 | Possible 3; none 14; definite 3 | Exact permutation | 12.232 | 0.002 | 0.009 | - |
| Depression severity | Definite 1; possible 12; normal 16 | Definite 0; possible 6; normal 14 | Exact permutation | 1.532 | 0.634 | 0.634 | - |

## Supplementary Table 1B. HTG

| **Measure** | **MoCA screen-positive** | **MoCA screen-negative** | **Test** | **Statistic/OR** | **Raw P** | **FDR q** | **AUC** |
| --- | --- | --- | --- | --- | --- | --- | --- |
| HAMA total | 9.0 [6.0, 12.0] | 6.0 [4.0, 10.0] | Mann-Whitney | 281.000 | 0.118 | 0.188 | 0.646 |
| HAMA somatic | 4.0 [2.0, 5.0] | 2.0 [1.0, 3.0] | Mann-Whitney | 307.000 | 0.026 | 0.052 | 0.706 |
| HAMD total | 8.0 [8.0, 11.5] | 9.0 [5.0, 11.0] | Mann-Whitney | 223.000 | 0.901 | 0.901 | 0.513 |
| PSQI total | 11.0 [7.0, 12.0] | 5.0 [4.0, 8.0] | Mann-Whitney | 343.500 | 0.002 | 0.014 | 0.790 |
| Anxiety status | 11/15 (73.3%) | 10/29 (34.5%) | Fisher | 5.225 | 0.025 | 0.052 | 0.694 |
| Depression status | 6/15 (40.0%) | 15/29 (51.7%) | Fisher | 0.622 | 0.535 | 0.671 | 0.441 |
| Anxiety severity | Possible 11; none 4; definite 0 | Possible 8; none 19; definite 1 | Exact permutation | 8.681 | 0.010 | 0.039 | - |
| Depression severity | Definite 0; possible 6; normal 9 | Definite 2; possible 13; normal 14 | Exact permutation | 1.348 | 0.587 | 0.671 | - |

*Scores are median [IQR]; categorical results are n/N (%). Exact fixed-margin permutation chi-square tests replace Monte Carlo estimates. FDR was controlled separately within subtype.*

# Supplementary Table 2. Vision-reduced and continuous MoCA analyses

| **Outcome** | **Adjustment** | **Estimate (95% CI)/groups** | **Effect** | **P value** |
| --- | --- | --- | --- | --- |
| MoCA-22 distribution | Unadjusted | NTG 18 [16, 19] vs HTG 19 [17.75, 20] | U=817.5 | 0.043 |
| Continuous MoCA-22 | Age + sex + education + VFI + RNFL | -0.82 (-1.53 to -0.11) | beta for NTG | 0.024 |
| Continuous MoCA-22 | Base + diabetes + systolic BP | -0.88 (-1.63 to -0.14) | beta for NTG | 0.020 |
| Continuous corrected MoCA | Age + sex + education + VFI + RNFL | -0.89 (-1.79 to 0.00) | beta for NTG | 0.050 |
| Continuous corrected MoCA | Base + diabetes + systolic BP | -1.07 (-1.98 to -0.16) | beta for NTG | 0.021 |
| MoCA-22 <18 | Crude; 23/49 vs 11/44 | 2.65 (1.10-6.42) | OR | 0.030 |
| MoCA-22 <18 | Age + sex + education + VFI + RNFL | 3.94 (1.24-12.49) | OR | 0.020 |

*MoCA-22 removes visuospatial/executive and naming (8 visually dependent points). Continuous models use HC3 robust standard errors. The <18 threshold is exploratory and not the primary endpoint.*

# Supplementary Table 3. Strict-IOP, interaction, and common-covariate analyses

| **Analysis** | **Term/model** | **Adjustment** | **Estimate (95% CI)** | **P value** |
| --- | --- | --- | --- | --- |
| Strict IOP >=25 | Model 0 | Crude | 2.79 (1.16-6.72) | 0.022 |
| Strict IOP >=25 | Model 1 | Age + sex + education | 7.40 (2.01-27.21) | 0.003 |
| Strict IOP >=25 | Model 2 | Model 1 + VFI | 6.68 (1.78-25.00) | 0.005 |
| Strict IOP >=25 | Model 3 | Model 2 + RNFL | 5.85 (1.43-23.90) | 0.014 |
| Strict IOP >=25 | Model 4 | Model 3 + diabetes | 6.38 (1.42-28.65) | 0.016 |
| Strict IOP >=25 | Model 5 | Model 4 + systolic BP | 6.74 (1.44-31.41) | 0.015 |
| Subtype interaction | PSQI | Age + sex + education + PSQI + RNFL | 1.11 (0.74-1.68) | 0.604 |
| Subtype interaction | RNFL | Age + sex + education + PSQI + RNFL | 0.77 (0.58-1.03) | 0.082 |
| Subtype interaction | Study-defined history of low blood pressure | Not estimable: equivalent history data were not collected for HTG participants | - | - |
| Common Firth model, NTG | PSQI | Age + sex + education + PSQI + RNFL | 1.32 (1.01-1.72) | 0.040 |
| Common Firth model, NTG | RNFL | Same | 0.77 (0.61-0.98) | 0.031 |
| Common Firth model, HTG | PSQI | Age + sex + education + PSQI + RNFL | 1.18 (0.91-1.53) | 0.213 |
| Common Firth model, HTG | RNFL | Same | 0.95 (0.87-1.04) | 0.271 |

*Strict IOP analysis excludes six HTG values (22, 22, 23, 23, 24.8, 24.8 mmHg), leaving n=38. The complete strict-IOP sequence now includes Models 0-5; Model 4 adds diabetes before Model 5 adds systolic blood pressure. Conventional interactions are primary; Firth interaction sensitivity P values were 0.612 (PSQI) and 0.092 (RNFL).*

# Supplementary Table 4. RNFL-visual-field structure-function correlations

| **Cohort** | **Pair** | **Spearman rho** | **P value** | **FDR q** |
| --- | --- | --- | --- | --- |
| Overall | RNFL-VFI | 0.482 | <0.001 | <0.001 |
| Overall | RNFL-MD | 0.419 | <0.001 | <0.001 |
| Overall | RNFL-PSD | -0.495 | <0.001 | <0.001 |
| NTG | RNFL-VFI | 0.274 | 0.057 | 0.170 |
| NTG | RNFL-MD | 0.200 | 0.169 | 0.254 |
| NTG | RNFL-PSD | -0.139 | 0.342 | 0.342 |
| HTG | RNFL-VFI | 0.655 | <0.001 | <0.001 |
| HTG | RNFL-MD | 0.607 | <0.001 | <0.001 |
| HTG | RNFL-PSD | -0.732 | <0.001 | <0.001 |

*Spearman correlations use bilateral mean RNFL and visual-field indices. FDR was controlled across VFI, MD, and PSD within each cohort stratum.*

# Supplementary Table 5. PSQI, RNFL, low blood pressure, and alternative-model sensitivities

| **Analysis** | **Model/definition** | **Estimate/counts (95% CI)** | **P value** |
| --- | --- | --- | --- |
| PSQI mood adjustment | Base NTG Firth model | 1.43 (1.03-1.98) | 0.031 |
| PSQI mood adjustment | + HAMA | 1.39 (1.01-1.93) | 0.046 |
| PSQI mood adjustment | + HAMD | 1.46 (1.02-2.09) | 0.038 |
| PSQI mood adjustment | + HAMA + HAMD | 1.47 (1.00-2.16) | 0.048 |
| RNFL clinical scale | Overall, per 5 micrometers thinner | 1.86 (1.15-3.02) | 0.011 |
| RNFL clinical scale | NTG, per 5 micrometers thinner | 5.86 (1.43-24.08) | 0.014 |
| RNFL clinical scale | HTG, per 5 micrometers thinner | 1.55 (1.01-2.38) | 0.043 |
| Low-blood-pressure counts | NTG study-defined low-blood-pressure history | 17/29 vs 4/20; crude OR 5.67 | 0.009 |
| Low-BP history ascertainment | Historical/medical-record criterion in all NTG | 49/49 assessed; 21/49 positive | - |
| Supplementary multi-time-point BP | Alongside 24-hour IOP; not used for classification | 27/49 overall; 13/21 among positive | - |
| Low-blood-pressure counts | Conventional study-visit seated definition | NTG 6/49; HTG 1/44 | 0.115 |
| Alternative confounding | VFI replaced by MD in final model | 7.96 (1.70-37.29) | 0.008 |
| Alternative confounding | Base visual model + BCVA | 7.18 (1.76-29.25) | 0.006 |
| Alternative confounding | Base visual model + CCT | 7.73 (1.83-32.63) | 0.005 |

*The base NTG Firth model adjusted for age, sex, education, PSQI, and RNFL. PSQI mood-adjustment rows are exploratory NTG Firth models. Study-defined history of low blood pressure and conventional study-visit seated hypotension are distinct variables. The history variable was ascertained only in NTG and required at least three resting seated upper-arm measurements on separate dates at systolic blood pressure <110 mmHg and/or diastolic blood pressure <65 mmHg from reported history or verifiable medical records. All 49 NTG participants were assessed under this criterion. Repeated multi-time-point measurement alongside 24-hour IOP monitoring was available for 27/49 and was contextual only; it did not determine classification. All 21 positive participants met the historical/record criterion, including 13 who also had multi-time-point measurement and eight who did not. Conventional study-visit seated hypotension used <90/<60 mmHg. Alternative confounding models assess robustness of the NTG-versus-HTG association.*
